# Analysis of glycation induced protein cross-linking inhibitory effects of some antidiabetic plants and spices

**DOI:** 10.1186/s12906-015-0689-1

**Published:** 2015-06-09

**Authors:** Handunge Kumudu Irani Perera, Charith Sandaruwan Handuwalage

**Affiliations:** Department of Biochemistry, Faculty of Medicine, University of Peradeniya, Peradeniya, Sri Lanka; Postgraduate Institute of Science, University of Peradeniya, Peradeniya, Sri Lanka

**Keywords:** Glycation, Cross-links, Inhibitors, SDS-PAGE, Antidiabetic plants, Spices

## Abstract

**Background:**

Protein cross-linking which occurs towards the latter part of protein glycation is implicated in the development of chronic diabetic complications. Glycation induced protein cross-linking inhibitory effects of nine antidiabetic plants and three spices were evaluated in this study using a novel, simple, electrophoresis based method.

**Methods:**

Methanol extracts of thirteen plants including nine antidiabetic plants and three spices were used. Lysozyme and fructose were incubated at 37 °C in the presence or absence of different concentrations of plant extracts up to 31 days. Standard glycation inhibitor aminoguanidine and other appropriate controls were included. A recently established sodium dodecyl polyacrylamide gel electrophoresis (SDS-PAGE) method was used to detect the products of protein cross-linking in the incubation mixtures.

**Results:**

High molecular weight protein products representing the dimer, trimer and tetramer of lysozyme were detected in the presence of fructose. Among the nine antidiabetic plants, seven showed glycation induced protein cross-linking inhibitory effects namely *Ficus racemosa* (FR) stem bark, *Gymnema sylvestre* (GS) leaves*, Musa paradisiaca* (MP) yam, *Phyllanthus debilis* (PD) whole plant, *Phyllanthus emblica* (PE) fruit, *Pterocarpus marsupium* (PM) latex and *Tinospora cordifolia* (TC) leaves*.* Inhibition observed with *Coccinia grandis* (CG) leaves and *Strychnos potatorum* (SP) seeds were much low. Leaves of *Gymnema lactiferum* (GL), the plant without known antidiabetic effects showed the lowest inhibition. All three spices namely *Coriandrum sativum* (CS) seeds, *Cinnamomum zeylanicum* (CZ) bark and *Syzygium aromaticum* (SA) flower buds showed cross-link inhibitory effects with higher effects in CS and SA. PD, PE, PM, CS and SA showed almost complete inhibition on the formation of cross-linking with 25 μg/ml extracts.

**Conclusions:**

Methanol extracts of PD, PE, PM, CS and SA have shown promising inhibitory effects on glycation induced protein cross-linking.

**Electronic supplementary material:**

The online version of this article (doi:10.1186/s12906-015-0689-1) contains supplementary material, which is available to authorized users.

## Background

Global prevalence of diabetes mellitus is rising with a particularly high increase of prevalence in developing countries [1]. Diabetes is associated with serious chronic complications such as retinopathy, nephropathy and cardiovascular diseases. A non-enzymatic process known as glycation, is accelerated as a result of persistently elevated plasma glucose levels occur in diabetic patients. Protein glycation has a primary role in the development of chronic diabetic complications [2, 3]. Hyperglycaemia accelerates the formation of covalent adducts with various structural and functional proteins such as collagen and albumin through glycation. The cells such as vascular endothelial cells and renal mesangial cells express high levels of the glucose transporter 1 and are more susceptible for complications associated with hyperglycaemia [4].

Over a period of time, glycation products form a complex family of stable heterogeneous group of compounds called advanced glycation end-products (AGEs) causing irreversible structural and functional damage to the affected protein molecules. Through various interlinked pathways, AGEs result in endothelial dysfunction leading to vasoconstriction, change in the endothelial surface in to a procoagulant surface, chemotaxis of macrophages and increased phagocytosis of modified lipids culminating in the formation of atherosclerotic plaques leading to macrovascular complications. Interaction of AGEs with receptors for advanced glycation end products (RAGE) induces the synthesis and release of cytokines which mediate enhanced production of collagen, laminin, and fibronectin leading to fibrosis [5].

Some AGEs are associated with inter or intra molecular covalent cross-links leading to adverse effects [6]. Cross-linked AGEs can be divided into fluorescent cross-linked AGEs such as glucosepane, pentosidine, crossline, glyoxal-derived lysine dimer (GOLD), methylglyoxal lysine dimer (MOLD) and non-fluorescent cross-linked AGEs such as imidazolium dilysine, alkyl formyl glycosyl pyrrole cross-links and arginine-lysine imidazole cross-links [4]. Devastating effects occur when protein cross-links are formed in long-lived proteins. Collagen being a major component of the extra cellular matrix and the longest lived protein in higher animals becomes a key target of glycation induced cross-linking [4]. Collagen is a major component of the blood vessels and in the framework of most of the parenchymal organs. Collagen is continuously exposed to glucose in vascular and extra vascular fluids [4]. Glycation induced cross-linking results in decreased vascular elasticity and reduced vascular compliance [5]. Cross-linking increases the rigidity of collagen and reduces its flexibility and susceptibility to enzymatic digestion leading to thickening of basement membranes as observed in microvascular lesions such as nephropathy [4].

Inhibition of glycation induced protein cross-linking is one of the therapeutic approaches that delay or prevent the progression of diabetic complications. A number of compounds recognized with potential glycation inhibitory effects including the extensively studied aminoguanidine (AG), failed at clinical trials due to their side-effects [7]. Recent studies have highlighted the importance of using plants with antiglycation properties [8] which may offer gentle and safe means of managing glycation induced molecular damage. Plants with antidiabetic effects are being used in traditional medicine for thousands of years without side effects. Spices are consumed in the daily diet as additives to improve the flavour of the food. A few studies have reported antiglycation effects of some medicinal plants using expensive specialized techniques such as high performance liquid chromatography, mass spectrometry, fluorescence spectrometry and specific ELISA assays [9]. However, only a minor fraction of these medicinal plants are scientifically validated for glycation induced protein cross-linking inhibitory effects.

In this study, the glycation induced protein cross-linking inhibitory effects of nine antidiabetic plants and three spices were evaluated, using a novel, simple, electrophoresis based method [10].

## Methods

### Plant materials

#### Antidiabetic plants

Parts from ten plants including nine antidiabetic plants were collected from Ratnapura District, Sri Lanka, namely, *Coccinia grandis* (L.) J. Voigt (*Kowakka*) (CG) leaves, *Ficus racemosa* L. (*Attikka*) (FR) stem bark, *Gymnema lactiferum* (L.) R. Br. ex Schult (*Kurinnan*) (GL) leaves, *Gymnema sylvestre* (Retz.) R. Br. Ex Schult (*Masbedda*) (GS) leaves, *Musa* X *paradisiaca* L. (*Alu kesel*) (MP) yam, *Phyllanthus debilis* Klein ex Willd (*Pitawakka*) (PD) whole plant, *Phyllanthus emblica* L. (*Nelli*) (PE) fruit, *Pterocarpus marsupium* Roxb. (*Gammalu*) (PM) latex, *Strychnos potatorum* L. f. (*Ingini*) (SP) seeds and *Tinospora cordifolia* (Willd.) Hook.f & Thoms. (*Rasakinda*) (TC) leaves*.* Plants were authenticated (HKIP-CSH-2013-01 to HKIP-CSH-2013-10) and the voucher samples were deposited at the Royal Botanical Gardens, Peradeniya, Sri Lanka. Plant parts were cleaned, shade dried for approximately 1 week and powdered. Dried latex of *P. marsupium* was used without further processing.

#### Spices

Dried *Coriandrum sativum* (Coriander) (CS) seeds, *Cinnamomum zeylanicum* (Cinnamon) (CZ) bark and *Syzygium aromaticum* (Clove) (SA) flower buds were purchased as branded products from the open market.

#### Preparation of methanol extracts

Plant parts were cleaned and powdered using a grinder. Dry powder (10 g) was extracted three times with methanol (100 ml) using a sonicator. Methanol extract was filtered and the solvent was evaporated by rotary evaporator (Buchi RII) at 45-50 °C. Dry methanol extracts were stored at room temperature until further analysis. Extracts and PM latex were resuspended in phosphate buffer (pH 7.4) to the required working concentrations before the experiments. Plant concentrations used in the reaction mixture were 1 to 5 mg/ml for initial screening. Concentration was reduced up to 5 or 1 μg/ml to assess the cross-link inhibitory effect of extracts which have shown strong inhibitory effects.

#### Detection of glycation induced protein cross-linking

A method developed to detect the inhibitory effects of plant extracts on protein cross-linking was used [10]. Briefly, working solutions of chicken egg lysozyme (Sigma), fructose and plant extracts were prepared using 200 mM phosphate buffer (pH 7.4) containing 0.02 % sodium azide. Lysozyme and fructose (500 mM) was incubated in the presence of plant extracts for up to 31 days at 37 °C. Final concentration of the plant extracts used for initial screening was 5 mg/ml and the concentration was reduced to 100, 50, 25, 5 or 1 μg/ml with the extracts that showed higher cross-link inhibitory effects. Standard inhibitor AG (Sigma) [10 mM (1.1 mg/ml)] was included as the positive control. A control was prepared with buffer, lysozyme and fructose. Corresponding blanks for tests and controls were prepared without fructose. Aliquots were collected at intervals (three times during the first week (*i.e.* Day 1, 2, 6 or 7) and two times thereafter until day 31 (*i.e.* Day 10, 14, 17, 21, 26, 31) and stored at −40 °C until further analysis. These aliquots were analyzed for the appearance of high molecular weight products using sodium dodecyl polyacrylamide gel electrophoresis (SDS-PAGE). SDS-polyacrylamide gels (12 %) were prepared according to the standard Laemmli procedure [11]. Aliquots from the incubation mixtures were heated with the SDS sample buffer at 95 °C for 3 min, before loading to the gel [11]. Broad range molecular weight markers (10–225 kDa) (Promega) were used to assess the approximate size of the high molecular weight products. Electrophoresis was carried out using Enduro Vertical Gel Electrophoresis system- E2010-P according to the standard Laemmli method [11]. After separation at pH 8.6, protein bands were visualized by staining with Coomassie brilliant blue. Appearance of high molecular weight products of lysozyme was assessed by visual observation.

All the experiments were repeated three times or more.

## Results

### Percentage yield of plant extracts

Percentage yield of methanol extracts were CG 6.36, FR 45.36, GL 15.45, GS 13.00, MP 2.00, PE 33.33, PD 20.09, SP 27.48 and TC 6.39. Higher yield with methanol was observed with bark, fruit (with seed) and seed. Lowest yield was observed with MP yam.

### Detection of glycation induced protein cross-linking

**Fig. 1:**
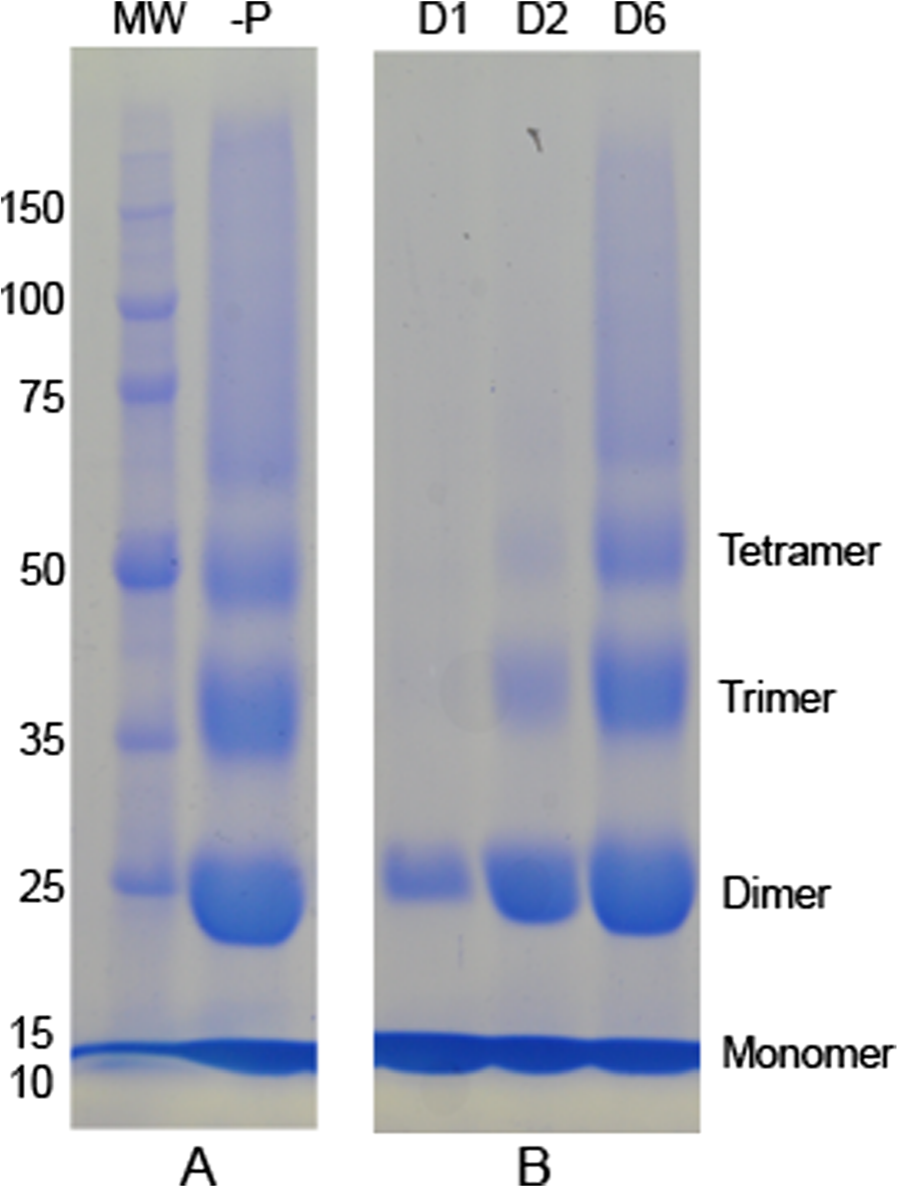
Formation of high molecular weight products in the presence of fructose. **a** Formation of protein cross-links in presence of fructose. SDS-PAGE was conducted with the aliquots collected at day 6. MW: Molecular weight markers, −P: in the presence of fructose without plant extracts or AG. This observation was made on varying incubation periods for more than 100 times. **b** Progress of protein cross-linking over time in the presence of fructose. SDS-PAGE was conducted with the aliquots collected at day 1 (D1), day 2 (D2) and day 6 (D6) from lysozyme incubated with fructose in absence of plant extracts or AG. Experiment was repeated three times

**Fig. 2:**
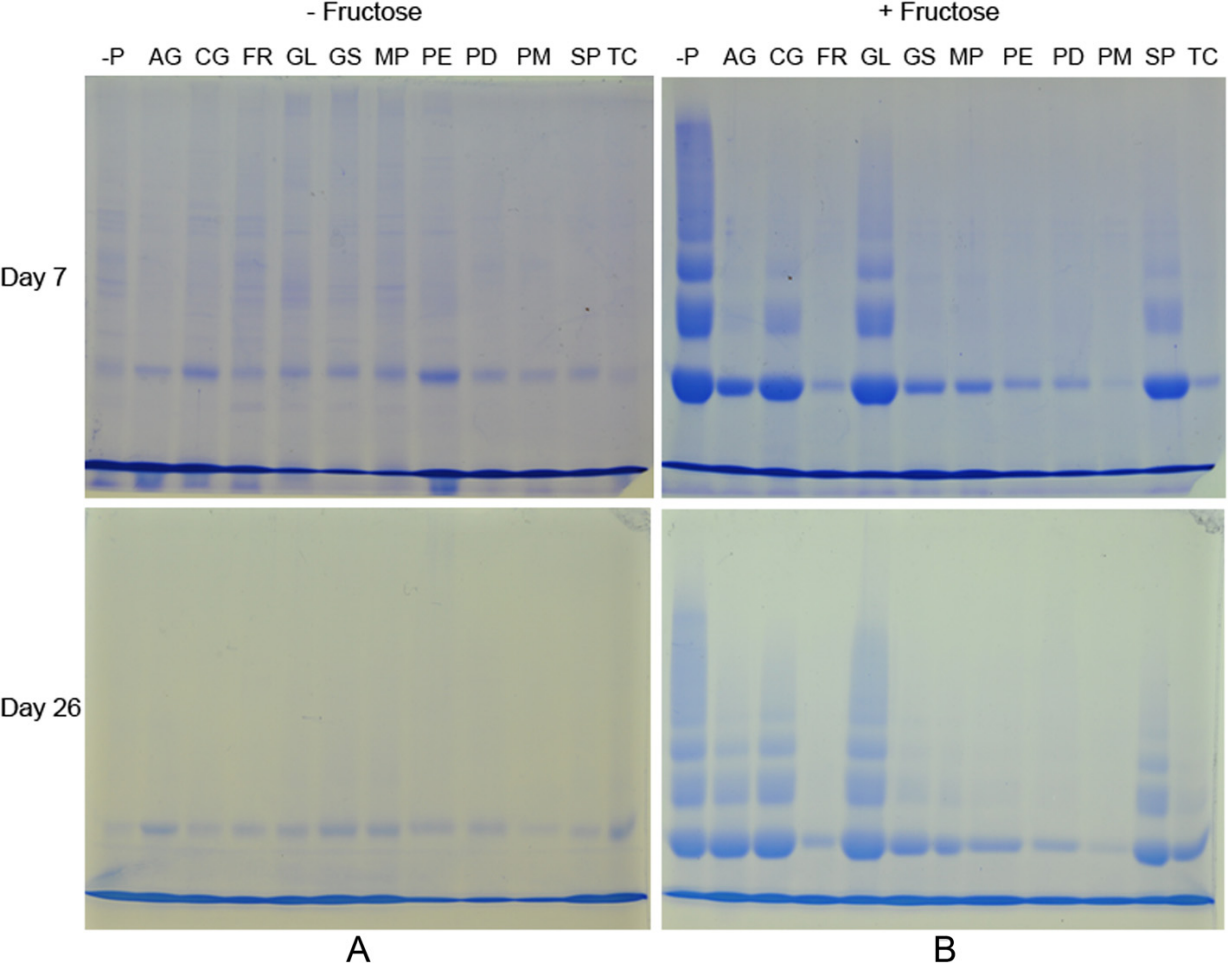
Effect of antidiabetic plants at 5 mg/ml on glycation induced protein cross-linking. SDS-PAGE was conducted using aliquots collected on day 7 (top) and day 26 (bottom). **a** Incubations carried out without fructose (−Fructose). **b** Incubations carried out with fructose (+ Fructose).  -P: in the absence of plant extracts or AG, AG: Aminoguanidine, CG: *C. grandis*, FR: *F. racemosa,* GL: *G. lactiferum,* GS: *G. sylvestre,* MP: *M. paradisiaca,* PE: *P. emblica,* PD: *P. debilis,* PM: *P. marsupium,* SP: *S. potatorum,* TC: *T. Cordifolia.* Experiment was repeated three times

**Fig. 3:**
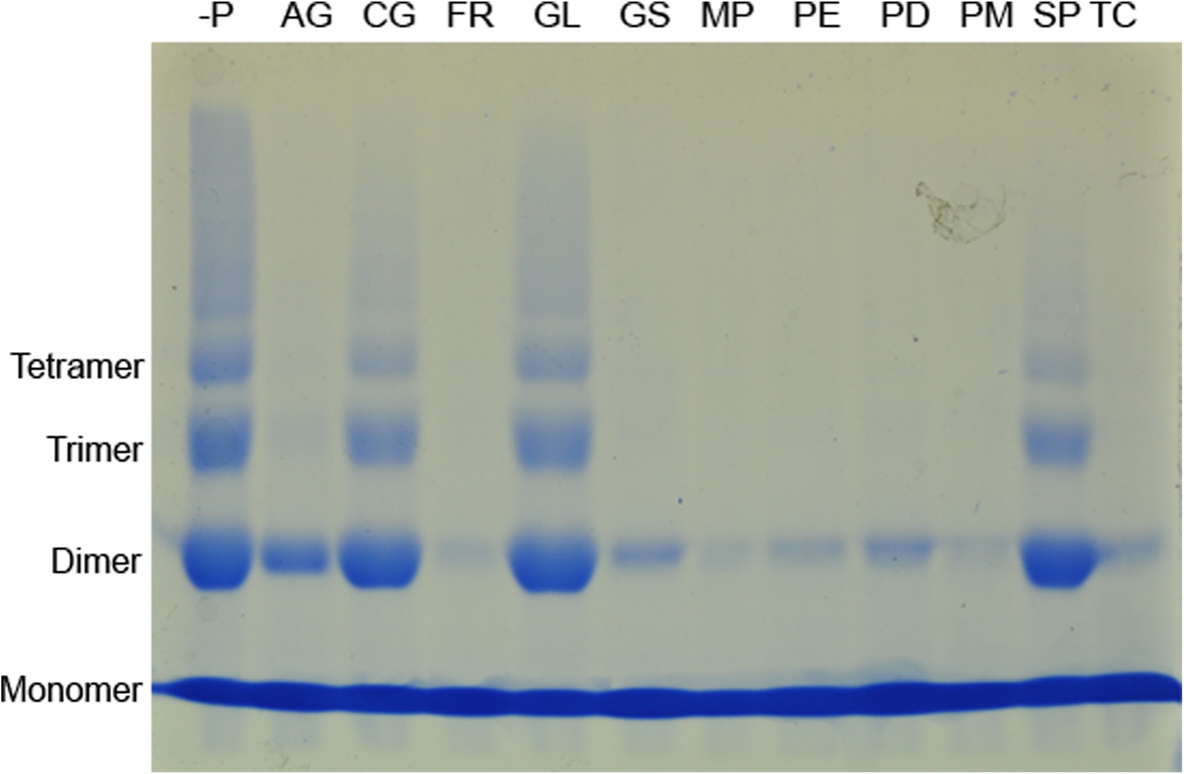
Effect of antidiabetic plants at 2.5 or 0.1 mg/ml on glycation induced protein cross-linking. SDS-PAGE was conducted using aliquots collected after 6 days. Incubations were carried out in the presence of fructose with different plant extracts. PE, PD and PM were used at 0.1 mg/ml. Rest of the plant extracts were used at 2.5 mg/ml. -P: in the absence of plant extracts or AG, AG: Aminoguanidine, CG: *C. grandis*, FR: *F. racemosa,* GL: *G. lactiferum,* GS: *G. sylvestre,* MP: *M. paradisiaca,* PE: *P. emblica,* PD: *P. debilis,* PM: *P. marsupium,* SP: *S. potatorum,* TC: *T. Cordifolia.* Experiment was repeated three times

**Fig. 4:**
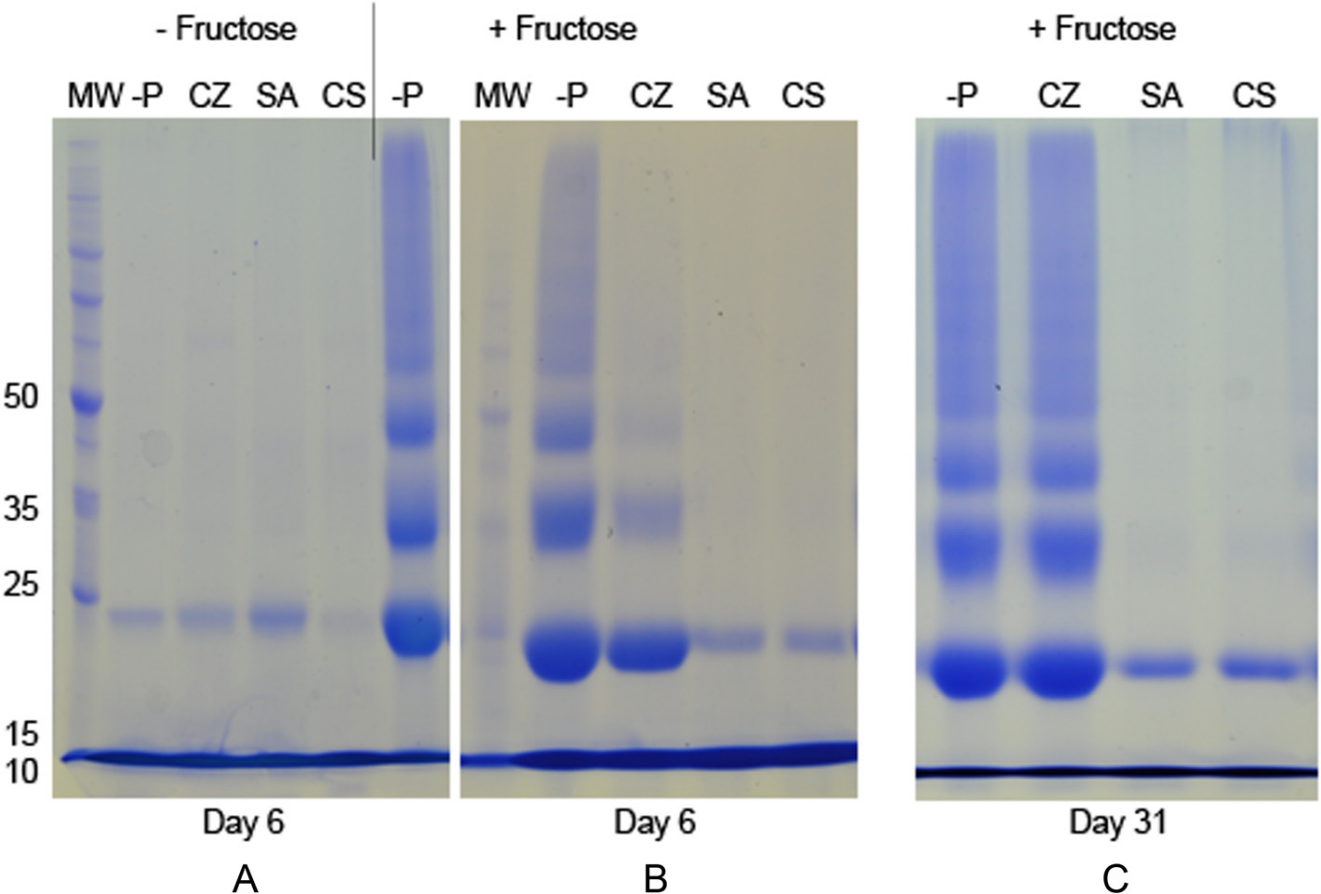
Effect of three spices on glycation induced protein cross-linking. SDS-PAGE was conducted using aliquots collected after 6 and 31 days. **​a** Incubations carried out without fructose (−Fructose) for 6 days. **b** Incubations carried out with fructose (+Fructose) for 6 days. **c**  Incubations carried out with fructose (+Fructose) for 31 days. Incubations were conducted in the absence of plant extracts or AG (−P) or presence of CS, CZ and SA (1 mg/ ml) CS: *C. sativum,* CZ: *C. zeylanicum,* SA: *S. aromaticum,* MW: Molecular weight markers. Experiment was repeated three times

**Fig. 5:**
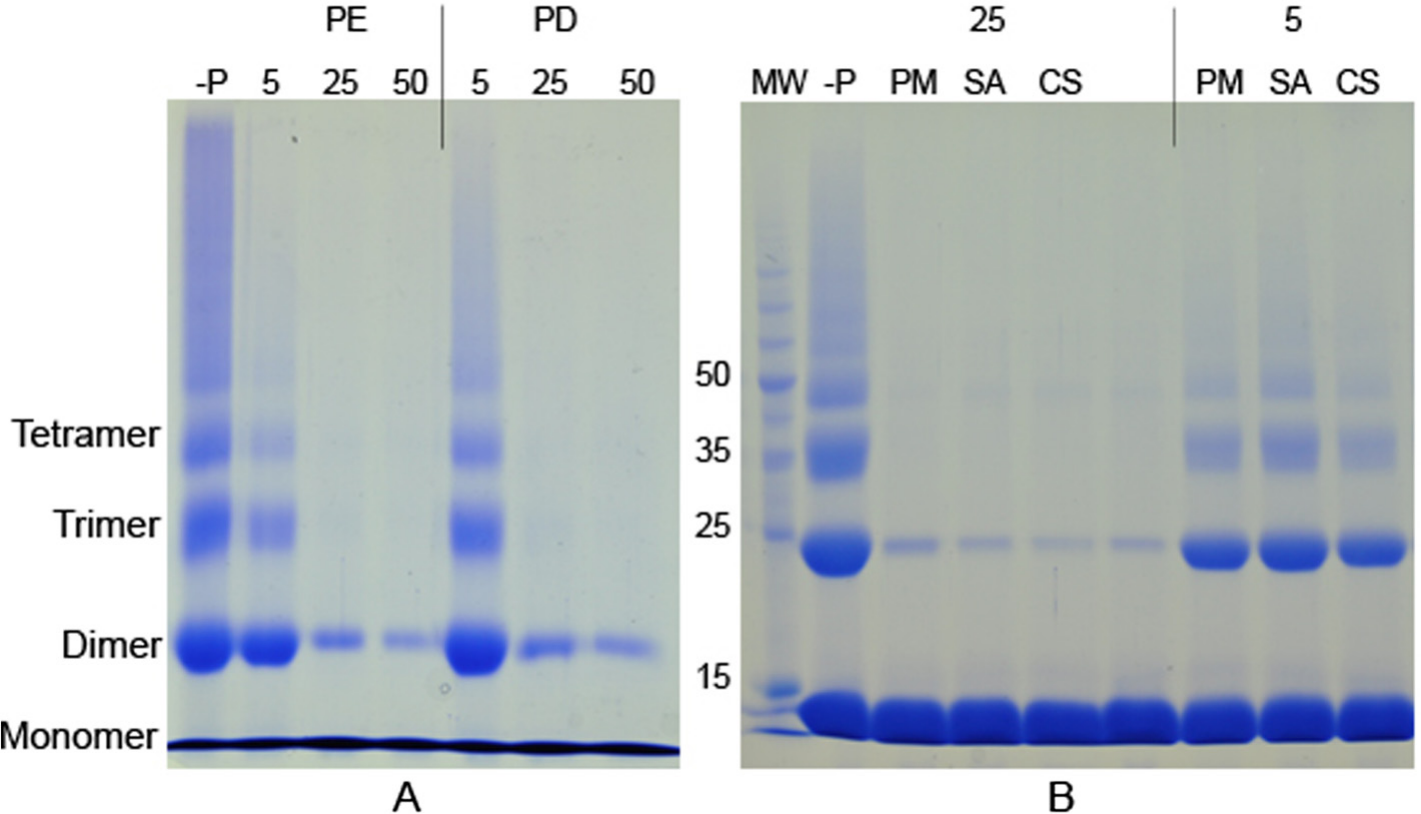
Effect of PE, PD, PM, CS and SA at lower concentrations on glycation induced protein cross-linking. **a**: Glycation induced protein cross-linking inhibitory potential of PE and PD. SDS-PAGE was conducted using aliquots collected after 17 days. Incubations were carried out in the presence of fructose without plant extracts or AG (−P) or with PE: *P. emblica* and PD: *P. debilis* at 5, 25 and 50 μg/ml. Experiment was repeated four times. **b**: Glycation induced protein cross-linking inhibitory potential of PM, CS and SA. SDS-PAGE was conducted using aliquots collected after 4 days (Additional file 1). Incubations were carried out in the presence of fructose without plant extracts (−P) or with 5 and 25 μg/ml, PM: *P. marsupium,* CS: *C. sativum* and SA: *S. aromaticum.* MW: Molecular weight markers. Experiment was repeated three times

SDS-PAGE of aliquots collected at various intervals of incubation in the presence or absence of plant extracts were compared. Molecular weight of the lysozme was approximately 12 KDa. High molecular weight protein products were formed when lysozyme was incubated with fructose (Figs. 1, 2, 3, 4, 5). Such products were not observed in the absence of fructose (Figs. 2, 4).

Cross-linked lysozyme was observed as high molecular weight bands. Those products represented the dimer (~24 KDa), trimer (~36 KDa) and tetramer (~48 KDa) of lysozyme when compared with the size of the unglycated lysozyme (~12 KDa) (Fig. 1a). Appearance of high molecular weight products gradually increased over time from day 1 to 6 (Fig. 1b). On day 1 only the dimer was observed (Fig. 1b).

### Detection of glycation induced protein cross-linking inhibitory potential of antidiabetic plants

In the absence of fructose (- Fructose) there was no increase in the appearance of high molecualr weight products (Fig. 2a). When compared with the unhibited control (−P), there was almost complete inhibition of glycation induced protein cross-linking in the presence of the extracts of FR, GS, MP, PD, PE, PM and TC during initial screening with 5 mg/ml extracts (Fig. 2b). This inhibition was observed on day 7 and persisted even at the later parts (Day 26) of the incubation (Fig. 2b). Lesser degree of inhibition of cross-linking was observed with 5 mg/ml extracts of CG and SP on day 7 (Fig. 2b). Amount of high molecular weight products observed with GL was close to that of uninhibited control (−P) indicating lack of inhibitory effects (Fig. 2b). Standard inhibitor AG showed almost complete inhibition of cross-linking on day 7 (Fig. 2b). However AG showed a lesser degree of inhibition of cross-linking on day 26 (Fig. 2b).

When 2.5 mg/ml extracts were used FR, GS, MP and TC showed almost complete inhibition (Fig. 3). These four extracts were not tested with lower concentrations in this study. CG and SP showed a low level of inhibition at 2.5 mg/ml. No inhibition was observed with GL at this concentration.

Lower concentrations of PD, PE and PM were used to investigate the inhibitory effects further. Almost complete inhibition of cross-linking was observed with 0.1 mg/ml extracts (Fig. 3). Inhibition of protein cross-linking was almost complete, even with 25 μg/ml PD, PE and PM showing evidence for strong glycation induced protein cross-linking inhibitory potential (Fig. 5). Reaction mixtures with 5 μg/ml extracts showed much lower inhibition (Fig. 5).

### Detection of glycation induced protein cross-linking inhibitory potential of spices

High molecular weight products of lysozyme appeared, when lysozyme was incubated in the presence of fructose (Figs. 4, 5). These changes did not occur in the absence of fructose (− Fructose) (Fig. 4a). In the presence of 1 mg/ml SA and CS, a marked inhibition of protein cross-linking was observed on day 6 (Fig. 4b). This inhibition was sustained even at 31 days (Fig. 4c). The inhibition observed with the CZ at 1 mg/ml was comparatively lower at day 6 (Fig. 4b). Extent of cross-linking observed in the presence of CZ on day 31 was similar to that of uninhibited control. This suggests the lack of sustainable inhibition with CZ on protein cross-linking (Fig. 4c).

Lower concentrations of CS and SA were used to investigate the inhibitory effects further. Protein cross-linking inhibition was almost complete, even with 25 μg/ml CS and SA (Fig. 5b). Reaction mixtures with 5 μg/ml extracts showed much lower inhibition (Fig. 5b).

## Discussion

Studies conducted to investigate protein glycation inhibitors have used expensive specialized equipments [9]. Previously we have optimized a simple SDS-PAGE method using lysozyme to detect the inhibitory effects of plant extracts on glycation induced lysozyme cross-linking [10]. Even though the glycation induced protein cross-linking inhibition by pure compounds has been studied using the SDS-PAGE method before [12], no reports were available for its application for crude plant extracts. Being a protein with a pI of 11.35 and having 6 Lysine residues and 11 Arginine residues which are the targets of glycation, lysozyme serves as a good candidate for glycation induced protein modifications. Lysozyme showed extensive cross-linking under the conditions that increase the protein glycation such as increase in the sugar concentration, incubation period and the reactivity of the sugar [10]. With SDS-PAGE, products of glycation induced cross-linking appeared as high molecular weight bands [10]. In the current study, the extent of cross-linking was compared by visual observation which gave only an idea about the approximate inhibition to the cross-link formation. A more accurate comparison would be achieved with quantification using a gel documentation system.

Methanol extracts were selected in the current study as they are known to contain negligible amounts of sugars which, if present, would interfere with the outcome of the glycation experiments. Antidiabetic plants selected in this study are being used to treat diabetes since ancient times and are proven for their hypoglycaemic properties experimentally too [13]. As per up-to-date literature, there are no previous reports available on the effect of these ten plants on glycation induced protein cross-linking even though PE fruit, MP inflorescence and TC bark were studied for their antiglycation effects. We have demonstrated cross-link inhibitory potential of the plants tested, using a relatively simple method established previously [10]. Resistant to cross-link formation (with no much variation from the respective blanks) was observed in the presence of extracts of seven antidiabetic plants demonstrating inhibitory effects. PD, PE, and PM demonstrated a strong inhibition to protein cross-link formation at a concentration as low as 25 μg/ml. Hence, our results suggest the presence of an additional beneficial effect of these antidiabetic plants which is likely to prevent chronic diabetic complications. However, these findings need to be confirmed using in vivo experiments. The effect of FR, GS, MP and TC at concentrations below 2.5 mg/ml was not evaluated in this study.

Previously we have analyzed antiglycation effects of the same extracts of nine antidiabetic plants and GL [14] using a different method in which bovine serum albumin (BSA) was taken as the model protein [15]. Our previous findings showed promising antiglycation effects in PD whole plant, PE fruit and PM latex [14]. Furthermore extracts of FR stem bark, GS leaf, MP yam and TC leaf also showed almost complete inhibition of glycation at 2 mg/ ml. Antiglycation effects observed with CG leaf and SP seed were much lower with a lowest inhibition observed with GL leaf. All those results on antiglycation effects tallied with the findings on protein cross-linking inhibitory effects of the present study. Therefore, when glycation products detected with PAGE are inhibited, protein cross-linking also is likely to be inhibited. The other possibility is that the extracts studied have inhibitory effects at early as well as late stages of glycation process. Fu *et al.,* revealed that cross-linking can be inhibited in the presence of metal chelators, sulfhydryl compounds, antioxidants and AG as they can uncouple glycation from subsequent cross-linking reactions [16]. Findings of Fu *et al.*, suggest that cross-link formation can be inhibited even after glycation is commenced.

As per up-to-date literature, there are no reports on glycation induced protein cross-link inhibitory effects or antiglycation effects of FR, GS, PD and SP other than our recent report on antiglycation effects [14]. Among these four plant parts, evidence for a strong glycation induced cross-link inhibitory effect was obtained for PD (whole plant) in the present study*.* Previously a strong in vitro antiglycation potential was revealed in PE fruit, with a reduction in fluorescent AGEs when BSA was used [17, 18]. Our results on cross-linking inhibition with PE support the findings of previous studies on its antiglycating effects. Different parts of MP and TC were studied previously on their antiglycation potential. MP (inflorescence) inhibited the formation of fluorescent AGEs [19]. TC stem extracts showed preventive effects on diabetic neuropathy in female Wistar rats [20]. Our results on cross-linking inhibition with MP yam and TC leaf support previous findings obtained on their antiglycating effects with different parts of the two plants.

When glycation process was studied under oxidative and antioxidative conditions using glucose and rat tail tendon collagen, it was shown that formation of glycoxidation products and cross-linking of collagen were inhibited under antioxidative conditions even though glycation was unaffected [16]. A good correlation has been shown between the free radical scavenging and antioxidant activities and glycation inhibitory activities of plant extracts [21, 22]. Previous studies have demonstrated the free radical scavenging and antioxidant activities of methanol extract of CG leaf [23], tannin fraction of FR bark [24], ethanol extract of FR bark [25], ethanol extract of GS leaf [26]*,* methanol extract of MP inflorescence [19], ethanol extracts of PE fruit [27, 28]*,* aqueous extract of PM stem bark [29], ethanol extract of SP seeds [30]‘ and methanol extract of TC stem [31] in vitro or in vivo. However, a proper comparison between the antioxidative effects of nine antidiabetic plants cannot be made using above data as the solvents used for extraction and the conditions used to measure the antioxidant effects were different.

Spices have contributed to the taste and flavor of foods for thousands of years. Although the beneficial effects of spices have been studied for some time on hypoglycaemic effects, studies carried out on the likely additional protective effects against diabetic complications are few. As per up-to-date literature, there are no reports on the effect of the CS and SA on glycation induced protein cross-linking. In one study, the aqueous extract of ginger, cinnamon, cumin, green tea, lemon, apple, garlic and black pepper were analyzed on their inhibitory effects on glycation induced protein cross-linking using eye lens soluble protein as the model protein instead of lysozyme [32]. They revealed 30–80 % inhibition of high molecular weight product formation with 1 mg/ml extracts, using SDS-PAGE. However the images of the gels were not included in this article [32].

We found evidence for strong glycation induced protein cross-linking inhibitory effects of CS and SA compared to that of CZ for the first time. Strong inhibition on protein cross-linking was observed with CS and SA at 25 μg/ml. Inhibition in the presence of CZ at 1 mg/ml was much lower compared to that of CS and SA and this inhibition did not sustain for a longer period. We have shown the inhibitory effects of CS previously at 5 mg/ml on the glycation induced protein cross-linking while establishing the method [8]. Results of the present study with SDS-PAGE method on cross-linking were tallied with the results we obtained on antiglycation effects of CS, CZ and SA [33], using a novel PAGE method and BSA [15].

Some of the spices are rich sources of antioxidants and hence they are beneficial against various diseases. The most potent antioxidant activities were found in 50 % ethanol extracts of cloves and cinnamon, when 24 herbs and spices were analyzed [21]. A positive correlation existed between the total phenolic content of the extract and the glycation inhibitory potential of spices [21].

A few studies showed the antiglycating effects of CS, CZ and SA using advanced equipment. One study reported the inhibitory effects on formation of fluorescent AGEs in the presence of ethanol extracts of CS, using spectrofluorimetry [34]. Nine other herbs and spices were included in their study. A major polyphenol isolated from CZ is the rutin which is known for its antiglycating effects [35]. In an earlier study, the best antiglycating effects were detected in cinnamon and clove among five culinary herbs and spices tested, which included rosemary, ginger and pepper [36]. Methanol extracts from eight dried spices namely, basil, cardamom, cinnamon, cumin, parsely, rosemary, thyme and turmeric, inhibited glycation of BSA incubated with glucose at various degrees with a high inhibition on formation of fluorescent AGEs with cinnamon [37]. Aqueous extract of clove inhibited the formation of fluorescent AGEs and CML, a biomarker of non florescent AGEs when BSA was incubated with 500 mM fructose [9]. This study has also demonstrated a potent antioxidant and free radical scavenging activity in the clove extract in consistent with the findings of the previous studies. Clove extract contains gallic acid, quercetin glucoside and ellagic acid among many polyphenolic compounds [38] which are known for their antiglycation effects [8]. Ellagic acid inhibited cross-linking of lysozyme in the presence of 400 mM ribose in a dose-dependent manner, as monitored by SDS-PAGE [12]. A group of researchers suggested that the hydroxyl and superoxide scavenging activity of clove which eventually reduce the dicarbonyl intermediates may be contributing to the glycation inhibitory effects [9].

Hypoglycaemic effects of CS, CZ and SA were previously shown in vivo using streptozotocin-induced diabetic rats, diabetic mice, or patients with type 2 DM [39–41]. The antiglycating effects observed with the plant extracts in our study were independent of their known hypoglycaemic effects, as the sugar concentrations used were similar in the presence and absence of the plant extracts. This suggests an additional beneficial effect of these extracts against glycation induced complications. Further studies are needed to investigate the efficacy and safety of these extracts in vivo.

## Conclusions

Using a novel, simple method we have demonstrated a strong glycation induced protein cross-linking inhibitory potential in *P. debilis* whole plant, *P. emblica* fruit, *P. marsupium* latex, *C. sativum* seed and *S. aromaticum* flower bud for the first time. These findings support the strong antiglycating effects we have demonstrated previously with these five plants parts and with *P. emblica, C. sativum* and *S. aromaticum* by others using different methods.

## Additional file

Additional file 1:
**Original gel of Fig.** 5b **.** Attached in response for the last comment of Referee 2.

## References

[1] Katulanda P, Ranasinghe P, Jayawardena R, Constantine GR, Sheriff MH, Matthews DR (2012). The prevalence, patterns and predictors of diabetic peripheral neuropathy in a developing country. Diabetology Metabolic Syndrome.

[2] Monnier VM. Glycation products as markers and predictors of the progression of diabetic complications. Annals of the New York Academy of Sciences. 2005;1043:567–581. 10.1196/annals.1333.065 16037280

[3] Goh SY, Cooper ME (2008). The role of advanced glycation end products in progression and complications of diabetes. J Clin Endocrinol Metabol.

[4] Singh VP, Bali A, Singh N, Jaggi AS (2014). Advanced glycation end products and diabetic complications. Korean J Physiology Pharmacol.

[5] Karmakar PS, Goswami RP (2012). Advanced glycation end products (AGEs): It’s role in the pathogenesis of diabetic complications. Med Update.

[6] Aronson D (2003). Cross-linking of glycated collagen in the pathogenesis of arterial and myocardial stiffening of aging and diabetes. J Hypertens.

[7] Rahbar S, Figarola JL (2003). Novel inhibitors of advance glycation end products. Arch Biochem Biophys.

[8] Wu C, Huang S, Lin J, Yen G (2011). Inhibition of advanced glycation end product formation by foodstuffs. Food Function.

[9] Suantawee T, Wesarachanon K, Anantsuphasak K, Daenphetploy T, Thien-Ngern S, Thilavech T (2014). Protein glycation inhibitory activity and antioxidant capacity of clove extract. J Food Sci Technol.

[10] Perera HKI, Ranasinghe HASK (2015). A simple method to detect plant based inhibitors of glycation induced protein cross-linking. Asian J Med Sci.

[11] Laemmli UK (1970). Cleavage of structural proteins during the assembly of the head of bacteriophage T4. Nature.

[12] Muthenna P, Akileshwari C, Reddy GB (2012). Ellagic acid, a new antiglycating agent: its inhibition of Nε-(carboxymethyl) lysine. Biochem J.

[13] Ediriweera ERHSS, Ratnasooriya WD (2009). A review on herbs used in treatment of diabetes mellitus by Sri Lankan ayurvedic and traditional physicians. Ayu.

[14] Perera HKI, Handuwalage CS (2015). Detection of protein glycation inhibitory potential of nine antidiabetic plants using a novel method. Asian J Med Sci.

[15] Wijetunge DCR, Perera HKI (2014). A novel in vitro method to detect inhibitors of protein glycation. Asian J Med Sci.

[16] Fu MX, Wells-Knecht KJ, Blackledge JA, Lyons TJ, Thorpe SR, Baynes JW (1994). Glycation, glycoxidation, and cross-linking of collagen by glucose: kinetics, mechanisms, and inhibition of late stages of the Maillard reaction. Diabetes.

[17] Povichit N, Phrutivorapongkul A, Suttajit M, Chaiyasut C, Leelapornpisid P (2010). Phenolic content and in vitro inhibitory effects on oxidation and protein glycation of some Thai medicinal plants. Pak J Pharm Sci.

[18] Perera PRD, Ekanayaka S, Ranaweera KKDS (2013). In vitro antiglycation activity of some medicinal plants used in diabetes mellitus. Medicinal Aromatic Plants.

[19] Nisha P, Mini S (2014). In vitro antioxidant and antiglycation properties of methanol extract and its different solvent fractions of *Musa paradisiaca* L. (cv Nendran) inflorescence. Int J Food Properties.

[20] Nadig PD, Revankar RR, Dethe SM, Narayanswamy SB, Aliyar MA (2012). Effect of *Tinospora cordifolia* on experimental diabetic neuropathy. Indian J Pharmacol.

[21] Dearlove RP, Greenspan P, Hartle DK, Swanson RB, Hargrove JL (2008). Inhibition of protein glycation by extracts of culinary herbs and spices. J Med Food.

[22] Elosta A, Ghous T, Ahmed N (2012). Natural products as anti-glycation agents: possible therapeutic potential for diabetic complications. Curr Diabetes Rev.

[23] Umamaheswari M, Chatterjee TK (2008). In vitro antioxidant activities of the fractions of *Coccinia grandis* L. leaf extract. Afr J Tradit Complement Altern Med.

[24] Sankaradoss N, Arun S, Naveen B, Sivanagamoorthi M, Velayudem R (2012). Antioxidant [In vitro] and analgesic activity [In vivo] of tannin fraction of stem bark of *Ficus racemosa* Linn. Res J Pharmaceutical, Biol Chem Sci.

[25] Veerapur VP, Prabhakar KR, Thippeswamy BS, Bansal P, Srinivasan KK, Unnikrishnan MK (2012). Antidiabetic effect of *Ficus racemosa* Linn. stem bark in high-fat diet and low-dose streptozotocin-induced type 2 diabetic rats: a mechanistic study. Food Chem.

[26] Rachh PR, Patel SR, Hirpara HV, Rupareliya MT, Rachh AS, Bhargava NM (2009). In vitro evaluation of antioxidant activity of *Gymnema sylvestre* r. br. leaf extract. Romanian J Biology Plant Biol.

[27] Rao TP, Sakaguchi N, Juneja LR, Wada E, Yokozawa T (2005). Amla (*Emblica officinalis Gaertn*.) extracts reduce oxidative stress in streptozotocin-induced diabetic rats. J Med Food.

[28] Kumaran A, Joel KR (2007). In vitro antioxidant activities of methanol extracts of five *Phyllanthus* species from India. Food Sci Technol.

[29] Mohammadi M, Khole S, Devasagayam TPA, Ghaskadbi SS (2009). *Pterocarpus marsupium* extract reveals strong in vitro antioxidant activity. Drug Discoveries Ther.

[30] Mishra SB, Verma A, Vijayakumar M (2013). Preclinical valuation of anti-hyperglycemic and antioxidant action of Nirmali (*Strychnos potatorum*) seeds in streptozotocin-nicotinamide-induced diabetic Wistar rats: A histopathological investigation. Biomarkers Genomic Med.

[31] Sivakumar V, Rajan MD (2010). Antioxidant effect of *Tinospora cordifolia* extract in alloxan-induced diabetic rats. Indian J Pharmaceutical Sci.

[32] Saraswat M, Reddy PY, Muthenna P, Reddy GB (2009). Prevention of non-enzymic glycation of proteins by dietary agents: Prospects for alleviating diabetic complications. Br J Nutr.

[33] Perera HKI, Wijetunge DCR (2015). Strong protein glycation inhibitory potential of clove and coriander. British J Pharmaceutical Res.

[34] Ramkissoon JS, Mahomoodally MF, Ahmed N, Subratty AH (2013). Antioxidant and antiglycation activities correlate with phenolic composition of tropical medicinal herbs. Asian Pacific J Tropical Med.

[35] Rao PV, Gan SH. Cinnamon: A Multifaceted Medicinal Plant. Evidence-Based Complementary and Alternative Medicine 2014, Article ID. 642942. 12 pages doi:10.1155/2014/642942 10.1155/2014/642942 PMC4003790 24817901

[36] Jin S, Cho KH (2011). Water extracts of cinnamon and clove exhibits potent inhibition of protein glycation and anti-atherosclerotic activity in vitro and in vivo hypolipidaemic activity in zebra fish. Food Chem Toxicol.

[37] Ho SC, Chang PW (2012). Inhibitory effects of several spices on inflammation caused by advanced glycation end products. American J Plant Sci.

[38] Atawodi S, Atawodi J, Pfundstein B, Spiegelhalder B, Bartsch H, Owen R (2011). Assessment of the polyphenol components and in vitro antioxidant properties of *Syzygium aromaticum* (L.) Merr and Perry. Electronic J Environmenta, Agricultural Food Chem.

[39] Eidi M, Eidi A, Saeidi A, Molanaei S, Sadeghipour A, Bahar M (2009). Effect of coriander seed (*Coriandrum sativum* L.) ethanol extract on insulin release from pancreatic beta cells in streptozotocin induced diabetic rats. Phytother Res.

[40] Sangal A (2011). Role of cinnamon as beneficial antidiabetic food adjunct: a review. Advances Applied Sci Res.

[41] Kuroda M, Mimaki Y, Ohtomo T, Yamada J, Nishiyama T, Mae T (2012). Hypoglycemic effects of clove (*Syzygium aromaticum* flower buds) on genetically diabetic KK-Ay mice and identification of the active ingredients. J Nat Med.

