# Supplementary figures and images for: Analysis of glycation induced protein cross-linking inhibitory effects of some antidiabetic plants and spices
Source: BMC Complement Altern Med. 2015 Jun 9;15:175. doi: 10.1186/s12906-015-0689-1 (PMC4459441; doi:10.1186/s12906-015-0689-1)

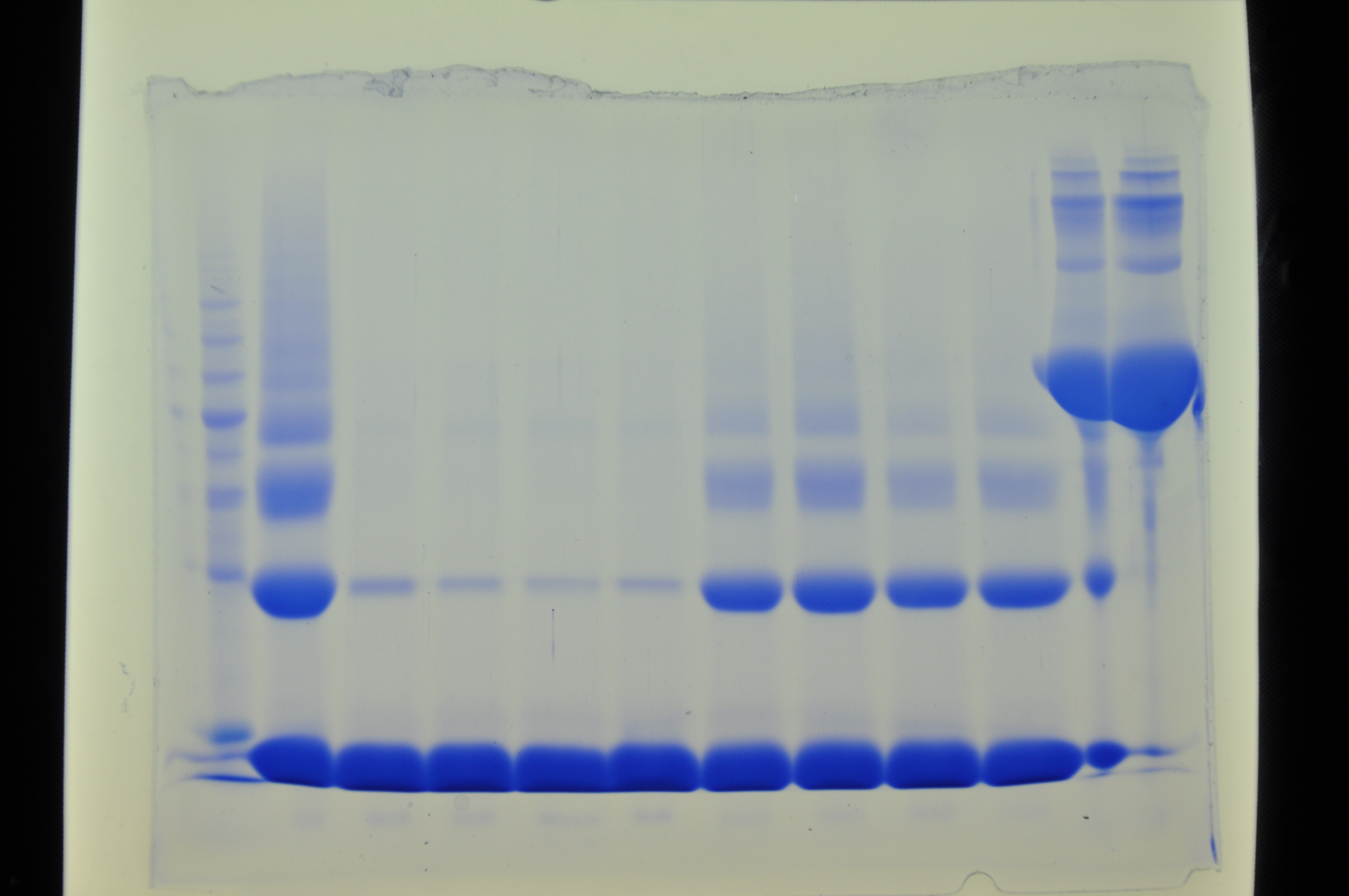

Supplement: Additional file 1: — Original gel of Fig. 5b . Attached in response for the last comment of Referee 2. [file 12906_2015_689_MOESM1_ESM.jpeg]
